# Identification of a novel *LAMA2* c.2217G > A, p.(Trp739*) mutation in a Moroccan patient with congenital muscular dystrophy: a case report

**DOI:** 10.1186/s12920-021-00959-2

**Published:** 2021-04-21

**Authors:** Youssef El Kadiri, Ilham Ratbi, Fatima Zahra Laarabi, Yamna Kriouile, Abdelaziz Sefiani, Jaber Lyahyai

**Affiliations:** 1 Centre de Recherche en Génomique des Pathologies Humaines (GENOPATH), Faculté de Médecine et de Pharmacie, Mohammed V University in Rabat, 10100 Rabat, Morocco; 2 Département de Génétique Médicale, Institut National d’Hygiène, BP 769 Agdal, 10090 Rabat, Morocco; 3 Unité de Neuropédiatrie et Maladies Neuro-Métaboliques, Service de Pédiatrie 2- Hôpital d’enfants, Rabat, Morocco

**Keywords:** *LAMA2* gene, Merosin-deficient congenital muscular dystrophy type 1A, NGS analysis, Nonsense mutation, Case report

## Abstract

**Background:**

Merosin-deficient congenital muscular dystrophy type 1A (MDC1A) is a rare autosomal recessive genetic condition caused by deleterious mutations in the *LAMA2* gene encoding the laminin-α2 chain. It is the most frequent subtype of congenital muscular dystrophies (CMDs) characterized by total laminin-α2 deficiency with muscle weakness at birth or in the first six months of life. To the best of our knowledge, this study reports the first molecular diagnosis and genetic defect of this heterogeneous form of CMD performed in a Moroccan medical genetic center using next-generation sequencing (NGS). It allows us to expand the mutational spectrum of the *LAMA2* gene.

**Case presentation:**

We report the case of a female Moroccan child with clinical and paraclinical features in favor of a CMD. She has global congenital hypotonia with generalized muscle weakness, psychomotor retardation, increased serum creatine kinase, and normal brain scan at the age of six months. Targeted NGS leads to the identification of a novel homozygous nonsense mutation c.2217G > A, p.(Trp739*) in the exon 16 of *LAMA2*. Sanger sequencing confirmed this mutation in the affected patient and showed that her parents are heterozygous carriers.

**Conclusions:**

A modern genetic analysis by NGS improves the genetic diagnosis pathway for adequate genetic counseling of affected families more precisely. An accession number from the National Center for Biotechnology Information (NCBI) ClinVar database was retrieved for this novel *LAMA2* mutation.

## Background

Laminin α2-related muscular dystrophy (*LAMA2* MD) is a rare autosomal recessive neuromuscular disorder caused by homozygous or compound heterozygous mutations in the *LAMA2* gene (MIM*156225) on chromosome 6q22-q23, encoding the laminin-α2 chain [1]. A total loss of merosin function due to pathogenic *LAMA2* mutations causes mainly a severe phenotype with early onset of merosin-deficient congenital muscular dystrophy type 1A (MDC1A). It is the most frequent form of congenital muscular dystrophy (CMD), responsible for 30% to 40% of all CMD cases in Europe [2]. MDC1A includes a broad range of phenotypes with different degrees of severity due to variability in the onset symptoms, clinical manifestations, and mutation effects on the laminin-α2 synthesis [3].

Herein, we report the case of a Moroccan female patient with a severe form of CMD caused by a novel homozygous nonsense mutation in the *LAMA2* gene identified by next-generation sequencing (NGS).

## Case presentation

**Fig. 1:**
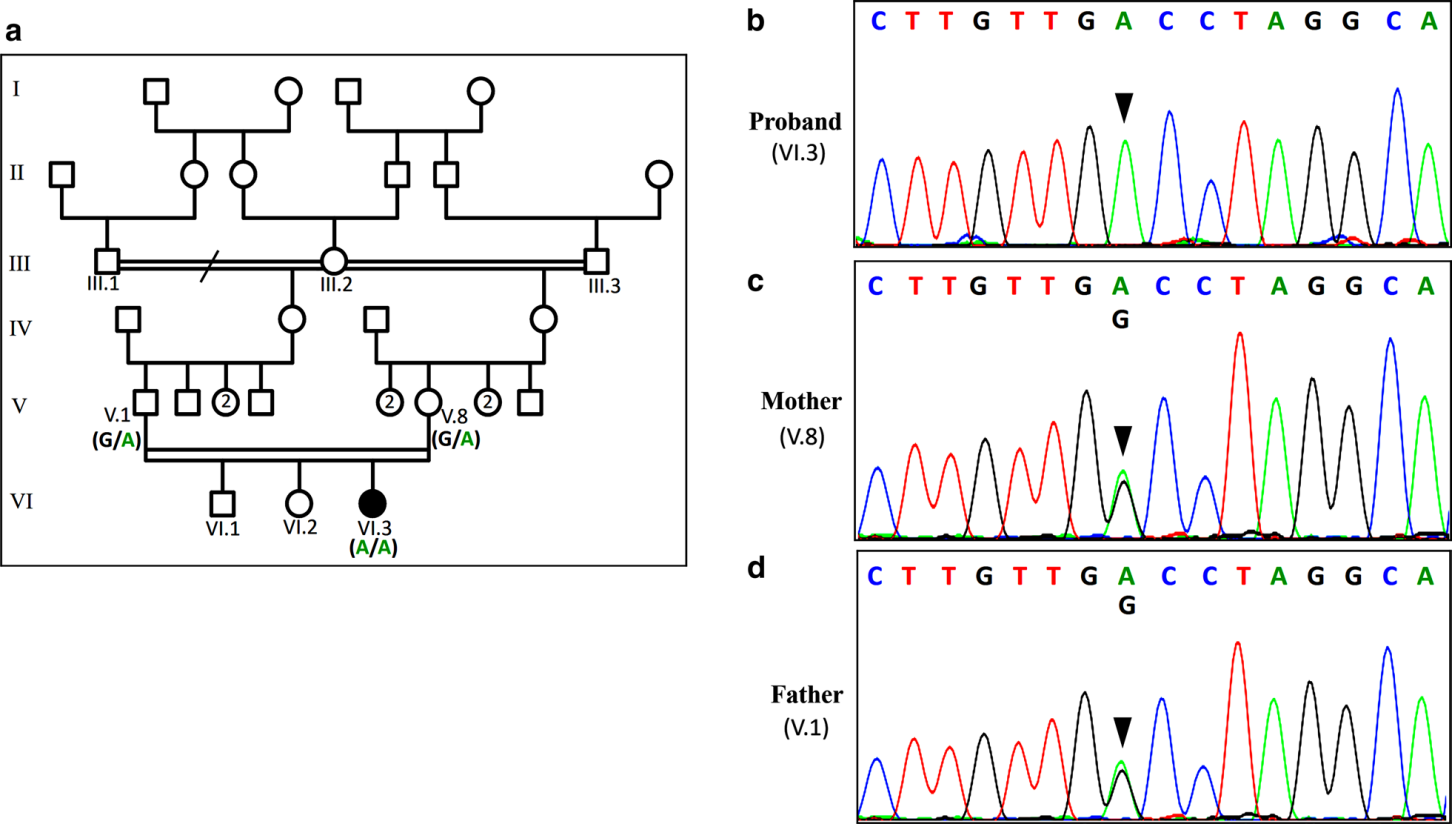
Pedigree of the Moroccan family with analysis of *LAMA2* mutation. (**a**) Pedigree of the studied family. The filled symbol represents the affected patient and open symbols represent unaffected individuals. The genotypes for the mutation c.2217G > A in the *LAMA2* gene are shown below the symbols V.1, V.8, and VI.3 (**b**) Electropherogram showing the c.2217G > A homozygous mutation in the proband. (**c**, **d**) Electropherograms showing the heterozygous mutation in both parents. Black arrows indicate homozygous and heterozygous mutation position detected in the proband and her parents, respectively

The proband (VI.3) is a female of 2 years and 7 months at the time of her genetic assessment. She is consanguineous, the third liveborn of a couple of half-first cousins (V.1 and V.8), sharing the same grand-mother (III.2). The grand-mother (III.2) was firstly married to her maternal cousin (III.1), grand-father of (V.1) and then to her paternal cousin (III.3), grand-father of (V.8). The proband’s siblings (VI.1 and VI.2) aged 10 and 6 respectively, were normal at the time of assessment. Grand-parents, parents, uncles, aunts and cousins of the proband (VI.3) were healthy with no particular disease history (Fig. 1a). The proband’s pregnancy was medically followed and presented without complication, but the baby was born prematurely at 33 weeks of gestation by vaginal delivery. Since the neonatal period, her parents have noticed a muscle weakness with hypotonia.

At 1 year of age, the neurologic evaluation showed a delay in motor development, tetraparesis with hypotonia, and an inability to sit. The tendon and bone reflexes were absent in lower limbs, and Babinski’s sign was negative. Her serum creatine kinase (CK) level was increased to 537 IU/l (normal range < 170 IU/l [4]). An electroneuromyographic analysis showed normal nerve conduction at the time of examination and the electromyogram (EMG) showed a myogenic pattern in muscles of upper and lower limbs. The computerized tomography (CT) scan at 6 months was normal and did not reveal any cerebral changes. Her intellectual development was normal and she never had seizures. She had no ocular signs and did not have cardiac nor respiratory dysfunction. She was unable to raise her head until 18 months and she was unable to stand up or stay up without support until now.

Clinical and paraclinical symptoms enable us to conclude that the patient’s phenotype may be compatible with a CMD. Thus, molecular genetic analysis was performed in the proband through a custom gene panel of 24 genes known to be related to inherited neuromuscular disorders in order to reach a precise molecular diagnosis (Table 1). The informed consent was obtained from the proband’s parents prior to molecular analysis. Samples of peripheral blood were collected from the affected child and her parents.

**Table 1:** Summary of neuromuscular disorders related genes included in the custom gene panel

Gene	Locus	Gene MIM number	Protein	Disease association	Mode of inheritance	Transcript
*CAPN3*	15q15.1	114,240	Calpain-3	LGMD	AD/AR	NM_000070.2
*COL6A1*	21q22.3	120,220	Collagen type VI alpha 1	CMD	AD/AR	NM_001848.2
*COL6A2*	21q22.3	120,240	Collagen type VI alpha 2	CMD	AD/AR	NM_001849.3
*COL6A3*	2q37	120,250	Collagen type VI alpha 3	CMD	AD/AR	NM_004369.3
*DMD*	Xp21.2	300,377	Dystrophin	DMD/BMD	XLR	NM_004006.2
*DNM2*	19p13.2	602,378	Dynamin 2	CM/CMD and other NMDs	AD/AR	NM_001005360.2
*DPM2*	9q34.13	603,564	Dolichyl-phosphate mannosyltransferase 2, regulatory subunit	CDG/CMD	AR	NM_003863.3
*EMD*	Xq27.3-q28	300,384	Emerin	EDMD	XLR	NM_000117.2
*FKRP*	19q13.32	606,596	Fukutin related protein	CMD/LGMD	AR	NM_024301.4
*FKTN*	9q31.2	607,440	Fukutin	CMD/LGMD	AR	NM_001079802.1
*ITGA7*	12q13	600,536	Integrin alpha-7	CMD	AR	NM_002206.2
*LAMA2*	6q22-q23	156,225	Laminin alpha-2	CMD/LGMD	AR	NM_000426.3
*LARGE*	22q12.3	603,590	Acetylglucosaminyltransferase-like protein	CMD	AR	NM_004737.4
*LMNA*	1q22	150,330	Lamin A/C	EDMD /CMD/LGMD and other NMDs	AD/AR	NM_170707.3
*PMM2*	16p13	601,785	phosphomannomutase 2	CDG	AR	NM_000303.2
*POMGNT1*	1p34.1	606,822	Protein O-linked mannose N-acetylglucosaminyltransferase-1 (beta 1,2-)	CMD/LGMD	AR	NM_001243766.1
*POMT1*	9q34.1	607,423	Protein O-mannosyltransferase 1	CMD/LGMD	AR	NM_007171.3
*POMT2*	14q24	607,439	Protein O-mannosyltransferase 2	CMD/LGMD	AR	NM_013382.5
*SEPN1/ SELENON*	1p36.13	606,210	Selenoprotein N1	CM/CMD/LGMD	AD/AR	NM_020451.2
*SGCA*	17q21	600,119	Alpha-sarcoglycan	LGMD	AR	NM_000023.2
*SGCB*	4q12	600,900	Beta-sarcoglycan	LGMD	AR	NM_000232.4
*SGCD*	5q33.3	601,411	Delta-sarcoglycan	LGMD	AR	NM_000337.5
*SGCG*	13q12-q13	608,896	Gamma-sarcoglycan	LGMD	AR	NM_000231.2
*TCAP*	17q12	604,488	Titin-Cap (Telethonin)	LGMD/CMD	AR	NM_003673.3

AD: autosomal dominant; AR: autosomal recessive; XLR: X linked recessive; LGMD: Limb-Girdle muscular dystrophy; CMD: Congenital muscular dystrophy; DMD: Duchenne muscular dystrophy; BMD: Becker muscular dystrophy; CM: Congenital myopathy; NMDs: Neuromuscular disorders; CDG: Congenital disorder of glycosylation; EDMD: Emery-Dreifuss muscular dystrophy. Cytogenetic location of genes was done according to the human reference genome-GRCh37 (hg19)

A DNA extraction was performed by the commercial PureLink™ Genomic DNA Mini Kit (Invitrogen-Thermo Fisher Scientific-USA). The quantity and quality of extracted genomic DNA (gDNA) were measured by a NanoDrop-2000 Spectrophotometer followed by Qubit 3.0 Fluorometer for more accurate DNA quantification using Qubit dsDNA HS (High Sensitivity) Assay Kit. In each sample, 10 ng of gDNA were used for multiplex PCR reactions using Ion Ampliseq™ On-Demand Primer Panel, which was designed via the Ion AmpliSeq™ Designer software (Thermo Fisher Scientific), available at (http://www.ampliseq.com). This allows highly multiplexed PCR amplification of 679 amplicons with superior coverage uniformity and specificity; in-silico covered 99% of regions of interest (ROI). Library construction was prepared using Ion AmpliSeq™ Library Kit v2.0 (Thermo Fisher Scientific), according to the manufacturer’s instructions. One of 16 barcodes of the Ion Xpress Barcode Adapters 1–16 Kit (Thermo Fisher Scientific) was added to each sample. The resulting DNA libraries were quantified with Qubit™ dsDNA BR Assay Kit on Qubit 3.0 Fluorometer (Molecular Probes) to get equimolar amounts of each library ready for downstream template preparation including clonal amplification by emulsion PCR that carried out on OneTouch™ 2 System (Thermo Fisher Scientific) with Ion PGM HI-Q™ view OT2 Kit (Thermo Fisher Scientific) coupled with the Ion OneTouch ES enrichment module (Thermo Fisher Scientific). Finally, the sequencing run was loaded on the 316™ Chip v2 (Thermo Fisher Scientific) using the Ion Torrent Personal Genome Machine (PGM, Thermo Fisher Scientific) through the Ion PGM HI-Q™ view Sequencing Kit as per the manufacturer’s protocol. Raw data were aligned to the hg19 human reference genome. Trimming, base calling, coverage analysis, and variant calling were performed on the Ion Torrent Server using the Torrent Suite software v.5.12.0 (Thermo Fisher Scientific), with default parameters.

Ion reporter software analysis v.5.10 (https://ionreporter.thermofisher.com/ir/) revealed a homozygous nonsense mutation in exon 16 of *LAMA2*, NM_000426.3(LAMA2):c.2217G > A (p.Trp739*). This mutation has never been reported in public human databases (accessed Nov 2020), including: ClinVar (https://www.ncbi.nlm.nih.gov/clinvar/), LOVD database (https://databases.lovd.nl/shared/genes/LAMA2), 1000 Genomes Project (https://www.internationalgenome.org/), gnomAD (https://gnomad.broadinstitute.org/), Exome Aggregation Consortium (http://exac.broadinstitute.org/), dbSNP Database (https://www.ncbi.nlm.nih.gov/snp/), and Human Gene Mutation Database (HGMD) (http://www.hgmd.cf.ac.uk/ac/). It was also not found in an in-house database of 100 Moroccan exomes (personal data).

Confirmation of this mutation was performed by conventional Sanger sequencing in the patient and her parents, using the BigDye™ Terminator v3.1 Cycle Sequencing kit (Applied Biosystems, Thermo Fisher Scientific) and loaded on an ABI 3500 automated Genetic Analyzer (Applied Biosystems, Thermo Fisher Scientific). Exon 16 and its flanking regions were amplified using the set of primers (ex16F: 5′GCTTTGACAAGCACATTTGATG3′/ex16R: 5′GGTCCCCAGGGTAGTAGCTG3′). A direct nucleotide sequence analysis confirmed that the proband carried the mutation in a homozygous state (Fig. 1b). Both parents were heterozygous (Fig. 1c, d).

The accession number from ClinVar database, SCV001448189.1 (submitted December 07, 2020), was assigned to our novel mutation.

## Discussion and conclusions

The CMDs are a group of inherited muscle diseases, genetically and clinically heterogeneous, mainly with an autosomal recessive type of inheritance. They are manifested in early life or infancy [5]. Some severe forms of CMD can be fatal in infancy, whereas others may have a milder course and can survive into adulthood [6]. To date, there are at least 32 genetic forms of CMD that have been associated with mutations in more than 30 genes coding for structural and functional proteins of skeletal muscle fibers [7, 8]. MDC1A, is the most common subtype of CMD in Caucasian populations due to laminin-α2 defects, accounting for 30 to 40 percent of total cases [2, 9].

We report here the clinical and molecular data of a patient with a severe phenotype of MDC1A. The patient manifested congenital hypotonia, muscle weakness, and had serum CK level which was three times the upper normal value. Similar values of CK levels were found in two unrelated Tunisian families with the same phenotype severity as our case, which had 3–5 times the normal range [10].

At the age of 6 months, our patient had a normal cerebral function without any specific change, observed by the CT scan. In contrast, Oliveira et al. reported a case of an MDC1A patient with white matter changes (WMC) observed in the CT scan [11].

In previous studies, patients with *LAMA2* mutations, showed normal brain magnetic resonance imaging (MRI) when has been detected during the neonatal period or in the first 6 months of life [11–13]. However, white matter abnormalities have been shown in the later period of life except for the case of an Italian patient reported by Saredi et al. [14], who carried a compound heterozygous nonsense mutation with an atypical phenotype and nearly normal brain MRI performed at 16 years of age. Thus, in our case, we could not completely exclude the absence of WMC, as well as the first detection by CT, which was done at an early age, it was considered as a period in which the changes are not always visible even with MRI observation in some patients.

Because of clinical heterogeneity and overlap between the different forms of CMD, NGS technology may be more efficient to increase the molecular diagnostic success rate, bypassing immunohistochemistry, which is not always available like in the Moroccan context [15, 16].

To the best of our knowledge, we report here the first molecular study performed on a Moroccan female proband underlining a novel nonsense mutation in the *LAMA2* gene using an Ion Torrent PGM platform. Our mutation c.2217G > A may cause a complete deficit in laminin-α2 function due to a premature termination codon (PTC) at 739 amino acid residue. The current mutation has never been reported in public human databases.

To date, more than 400 different pathogenic mutations in the *LAMA2* gene have been reported in HGMD professional release 2019.4 (accessed Nov 2020), (http://www.hgmd.cf.ac.uk/ac/gene.php?gene=LAMA2), with no noticeable mutational hotspot. The most frequently reported genotype in the literature for patients with MDC1A were mutations that create a PTC associated with a total absence of laminin-α2 and detected in a homozygous state [11, 12, 17].

**Fig. 2:**
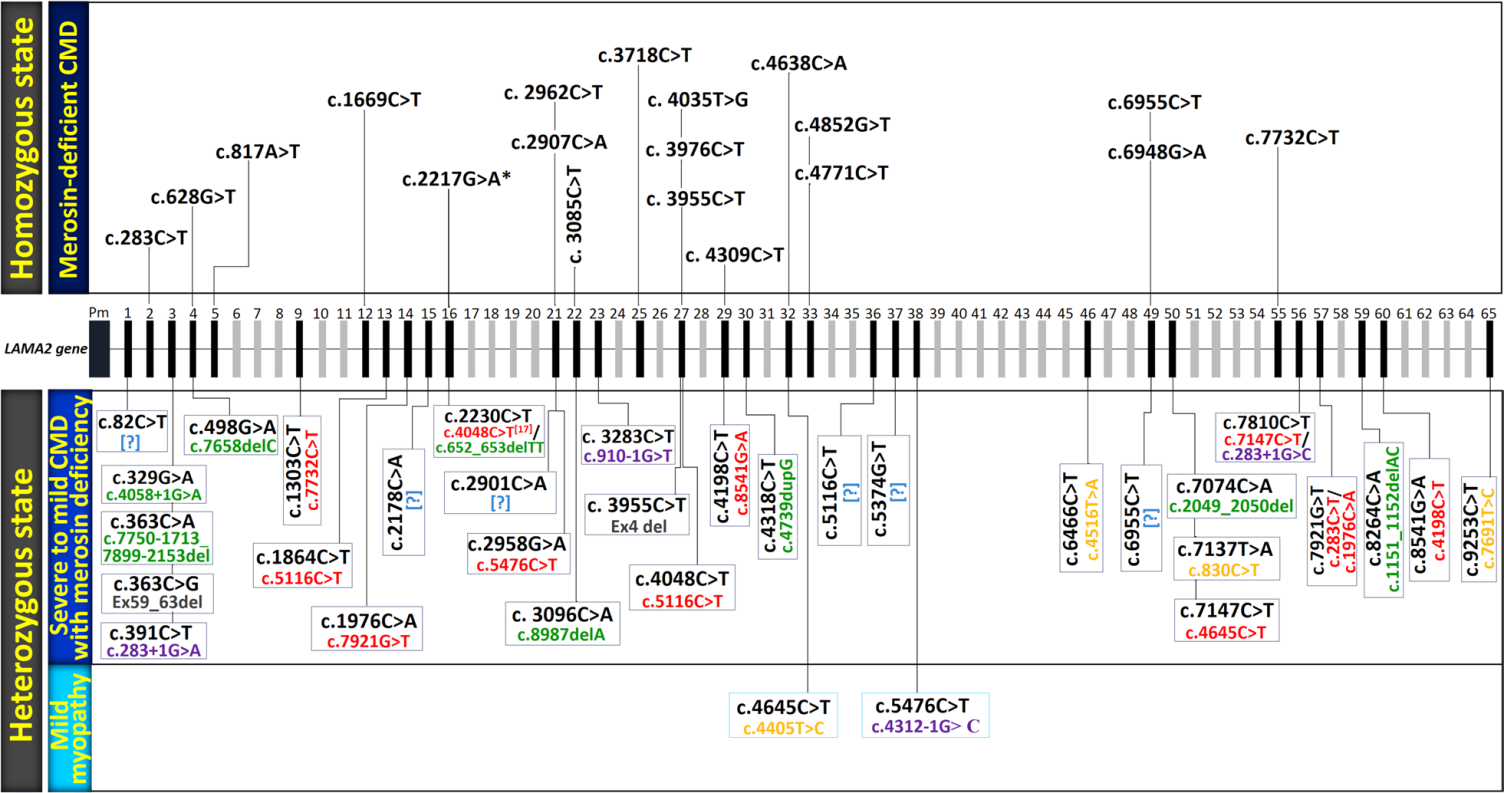
Schematic representation of the *LAMA2* gene structure and localization of identified *LAMA2* nonsense mutations that are listed in the public version of HGMD. Two classifications, homozygous and heterozygous state, of *LAMA2* nonsense mutations scattered along the coding sequence containing 65 exons (only those containing nonsense mutations are described and marked in black) are shown in patients with severe or milder phenotype of CMD (in dark blue) and patients with mild myopathy (in light blue). Nonsense mutations listed in HGMD are shown in black. Mutations of the second allele are illustrated in the following manner: nonsense (in red), frameshift (in green), missense (in yellow), splice site (in purple), and in-frame deletion (in grey). [?] refers to an unknown second mutation from the other allele due to using classical tests that could not identify the second mutation in the proband. The asterisk (*) refers to the novel mutation identified in our Moroccan patient. cDNA reference sequence that has been used to identify these mutations was NM_000426.3

We have illustrated in (Fig. 2), all pathogenic *LAMA2* nonsense mutations reported in the public version of HGMD in patients with severe merosin-deficient CMD or milder myopathy according to zygosity state. We observed that the effect of mutations on phenotype development and its severity differs between patients regardless of their mode of inheritance, which makes the understanding of the physiopathological mechanisms so far unknown. Interestingly, in the same exon 16, a nonsense mutation c.2230C > T (p.Arg744*) was previously reported at a homozygous state in two adult siblings with a mild MD phenotype [18]. Whereas the same mutation at compound heterozygous state together with another nonsense mutation c.4048C > T (p.Arg1350*) in exon 27 is responsible for causing a severe CMD [17].

In conclusion, NGS has revolutionized molecular genetic research and has become an essential genetic tool for molecular diagnosis of heterogeneous disorders as CMDs. It allows us to improve our understanding of the origin of MDC1A-causing by the identification of a mutation in the *LAMA2* gene. The novel mutation identified here provides an appropriate course of management to the patient to offer genetic counseling to the family, to expand the genetic spectrum of LAMA2-related CMDs, and to raise awareness to the pediatric neurologists on the morbidity of this severe form of CMD that is so far under-diagnosed in the Moroccan population, and to show them the added value of NGS technology in reducing diagnostic wandering.

## Data Availability

The principal data generated and/or analyzed during the current study are included in the published article. The datasets used in this study are available from the corresponding author on reasonable request.

## Abbreviations

MDC1A: Merosin-deficient congenital muscular dystrophy type 1A
CMD: Congenital muscular dystrophy
CK: Creatine kinase
NGS: Next-generation sequencing
MD: Muscular dystrophy
EMG: Electromyogram
CT: Computerized tomography
WMC: White matter changes
MRI: Magnetic resonance imaging
PTC: Premature termination codon
HGMD: Human gene mutation database
